# The Emotion Regulation Roots of Job Satisfaction

**DOI:** 10.3389/fpsyg.2020.609933

**Published:** 2020-11-24

**Authors:** Hector P. Madrid, Eduardo Barros, Cristian A. Vasquez

**Affiliations:** ^1^School of Management, Pontificia Universidad Católica de Chile, Santiago, Chile; ^2^School of Business, Universidad Adolfo Ibáñez, Santiago, Chile; ^3^Alliance Manchester Business School, The University of Manchester, Manchester, United Kingdom

**Keywords:** job attitudes, job satisfaction, emotion regulation, affect, diary study

## Abstract

Job satisfaction is a core variable in the study and practice of organizational psychology because of its implications for desirable work outcomes. Knowledge of its antecedents is abundant and informative, but there are still psychological processes underlying job satisfaction that have not received complete attention. This is the case of employee emotion regulation. In this study, we argue that employees’ behaviors directed to manage their affective states participate in their level of job satisfaction and hypothesize that employee affect-improving and -worsening emotion regulation behaviors increase and decrease, respectively, job satisfaction, through the experience of positive and negative affect. Using a diary study with a sample of professionals from diverse jobs and organizations, for the most part, the mediational hypotheses were supported by the results albeit a more complex relationship was found in the case of affect worsening emotion regulation. This study contributes to expanding the job satisfaction and emotion regulation literatures and informs practitioners in people management in organizations about another route to foster and sustain positive attitudes at work.

## Introduction

A central concern for organizations is to foster and sustain job satisfaction among their employees, which involves managing the conditions for building positive judgments about their work environment and organizational membership (Weiss, 2002). The relevance of job satisfaction relies on its effects on work-related outcomes, such as the intention to remain or leave the organization, together with desirable behavior embedded in contextual performance (Bowling and Hammond, 2008). As such, over a few decades, a weight of research has examined and supported a series of variables participating in the construction of this job attitude, including contextual variables and individual differences (Judge et al., 2017). For example, at the contextual level, antecedents of job satisfaction are job characteristics, group and team processes, supervision and leadership behavior, and human resource practices (Judge and Kammeyer-Mueller, 2012). Furthermore, job satisfaction is also a function of individual differences. Personality traits such as extroversion and neuroticism, together with positive and negative trait affect, are positively and negatively related to whether employees have positive judgments about their work (Judge et al., 2008). Therefore, today we have a fairly comprehensive understanding of what makes employees satisfied at work.

Notwithstanding, there are still issues not completely explored in this field of inquiry, particularly at the individual differences level of analysis. This is the case of the role that emotion regulation possibly plays in the experience of job satisfaction. Emotion regulation refers to the set of behaviors that individuals enact to manage, sustain, or change their affective experiences, which influences feeling and thinking (Gross, 1998). Thus, because job satisfaction has affective rudiments (Weiss and Cropanzano, 1996), the way employees manage their emotions might have an influence on this attitude. In this regard, the related construct of emotional intelligence has been studied about job satisfaction (Miao et al., 2017); however, studies are limited to general ratings about abilities for identifying, understanding, utilizing, and managing emotions (Mayer et al., 2008; Côté, 2014). Consequently, this stream of research has not informed us about the specific emotion regulation behaviors that might be associated with being satisfied at work.

This dearth of knowledge denotes a limitation in the job satisfaction literature because emotion regulation is part of regular behavior in the diverse domains of our lives, including the workplace (Gross and Thompson, 2007). Therefore, if emotion regulation participates in the construction of job satisfaction, there could be another way to facilitate the emergence of positive attitudes in organizations. Hence, this study aims to address these issues by arguing, and empirically examining, if specific behavioral strategies to improve or worsen own feelings increase and decrease job satisfaction, through the experience of positive and negative affective states, respectively. In this sense, it contributes both to the job satisfaction and the emotion regulation literatures. Specifically, this study extends our knowledge on behavioral processes, with affect meaning, that have the potential to drive employees’ judgments about their jobs and organizations. Furthermore, in a broader sense, it enriches our understanding of the effects and implications of specific emotion regulation behaviors directed not only at improving but also at worsening one’s own feelings in the workplace.

### Theoretical Development

Emotion regulation behavior is a core psychological function for human adaptation. It is through the chance of changing or sustaining the affective experience that we cope with environmental demands and take advance of opportunities available in a given context (Gross, 1998). Thus, we often behave to modify the affective-laden situations where we are in, reappraise or distract from the affective-eliciting events confronted, and modulate emotions derived from these events (Gross, 2013). Accordingly, psychological research has been consistent in showing the benefits of emotion regulation for individual performance and well-being, in the workplace inclusive (Diefendorff et al., 2008; Lawrence et al., 2011). A comprehensive conceptual system about emotion regulation behavior at work is the Emotion Regulation of Others and the Self (EROS) model (Niven et al., 2011). Accordingly, individuals actively engage in actions to manage their affective experiences to improve or worsen their feelings. According to this model, affect-improving emotion regulation refers to behaviors involving cognitive reappraisal and attentional deployment of events experienced, to provoke or maintain positive feelings in the subjective realm. Cognitive reappraisal consists of modifying events’ meaning with a focus on their benefits and brighter side (Brans et al., 2013). In the case of attentional deployment, this self-regulation strategy conveys the detachment of attention from events triggering the current negative feelings, by means of focusing on less aversive aspects of the situation or on other positive images or previous experiences, consequently facilitating the management of psychological resources (Quoidbach et al., 2015). Furthermore, EROS holds that affect-worsening emotion regulation entails the series of behaviors that increase the feeling of negative affect. Thus, affect-worsening behaviors are a form of affective dysregulation expressed, for instance, in cognitive rumination, which refers to the individuals’ engagement in repetitive and rigid thinking about negative feelings and events occurring in their lives (Lyubomirsky and Nolen-Hoeksema, 1993; Treynor et al., 2003).

We argue that emotion regulation should play a role in the construction of job satisfaction, such that employees’ affect-improving and affect-worsening emotion regulation, by the experience of positive and negative affect, would respectively, increase and decrease positive attitudes toward the work and the organization. These proposals are in line with previous research on emotional work. Zapf (2002), reviewed the literature on emotional labor showing that strategies for expressing or suppressing emotion have a positive or negative impact on the sense of well-being, conveyed, for instance, in job satisfaction. In a similar vein, in the case of affect-improving emotion regulation, employees may transform the meaning of adverse and challenging work cognitions, improving the subjective denotation of their work situations and embedding events (cf., Brans et al., 2013; Katana et al., 2019; Madrid, 2020). Positive reframing increases the sense of well-being in the form of, for example, positive emotions (Lambert et al., 2009; Cortini et al., 2019). Furthermore, attentional deployment should take the individual away from work situations causing negative emotional reactions, thus facilitating the emergence of positive feelings (cf., Quoidbach et al., 2015). Hence, employees who are prone to use affect-improving emotion regulation strategies should experience positive affect more often in organizations. On the other hand, affect-worsening emotion regulation should make employees disposed to experience negative affect more frequently. This effect is probably due to the cognitive rumination, which may lead employees to concentrate on drawbacks, over opportunities, at work (Nolen-Hoeksema, 2000; Verhaeghen et al., 2005; Madrid et al., 2015).

Once positive and negative feelings emerge from affect-improving and worsening emotion regulation at work, they might be associated with job satisfaction. The affective experience has pervasive influences in the way we think and behave, such that, at the cognitive level, it infuses perception, memory, information processing, and, thereby, attitudes and judgments (Forgas, 1995). Positive feelings color perception and they make individuals inclined to attend the positive attributes of objects, people, or events met, and they also prime memory, such that individuals are disposed to recall events and memories congruent with the pleasure of the feelings experienced (Isen, 2008). Furthermore, when experiencing positive feelings, individuals process information flexibly and in a broader way, allowing them to build a more comprehensive view and understanding of their environment (Fredrickson, 2001).

Therefore, we argue that employees that often experience positive affect, due to affect-improving regulation, should be more focused on the brighter side of their work context, make inferences based on memories about successful work events and look at the organizational environment with a flexible and broader approach. As a result, they should have a more favorable judgment about the work and the organization, namely, higher job satisfaction. In turn, the opposite are the effects of negative feelings on cognition (Schwarz and Skurnik, 2003; Gable and Harmon-Jones, 2010). In this case, negative affect directs the attention to the unfavorable elements in the environment, primes memory toward displeasing and unsuccessful events and experiences, and leads to a more convergent and narrower way of thinking (Nolen-Hoeksema, 2000). Accordingly, it might be expected that under this state, emerging from affect-worsening regulation, employees feel more dissatisfied because they may be biased toward the unfavorable conditions available in the work environment, dismissing those rewarding elements of the organizational context.

Taking the above together, we propose that the relationship between emotion regulation behavior, affect and job satisfaction unfolds through two mediational processes:

Hypothesis 1: Affect-improving emotion regulation will be positively related to positive affect, which in turn will be positively related to job satisfaction, such that positive affect will mediate the relationship between affect-improving emotion regulation and job satisfaction.
Hypothesis 2: Affect-worsening emotion regulation will be positively related to negative affect, which in turn will be negatively related to job satisfaction, such that negative affect will mediate the relationship between affect-worsening emotion regulation and job satisfaction.

The effects of emotion regulation on job satisfaction are expected to occur over and above trait affect. The latter are temperamental dispositions conveying the tendency to feel and react to the environment with positive and negative feelings (trait positive and negative affect, respectively) (Watson, 2000), which, according to previous research, are antecedents of job satisfaction (Judge et al., 2008). Thus, we expect that emotion regulation behavior would exert an incremental effect on job satisfaction over the employees’ personality.

Means, standard deviations, reliabilities, and correlations are presented in Table 1.

**TABLE 1:** Means, standard deviations, correlations, and reliabilities.

Variable	*M*	SD	1	2	3	4	5	6	7	8
1. Professional role (0 = No, 1 = Yes)	0.27	0.45	–							
2. Positive trait affect	3.76	0.63	0.06	**(0.80)**						
3. Negative trait affect	2.17	0.73	0.11	−0.30*	**(0.83)**					
4. Affect-improving regulation	3.84	0.62	0.10	0.32*	−0.18	**(0.72)**				
5. Affect-worsening regulation	1.24	0.47	0.12	−0.30*	0.38**	−0.01	**(0.83)**			
6. Positive affect	3.55	0.68	−0.12	0.56**	−0.33*	0.40**	−0.27*	**(0.84)**	−0.14**	0.32**
7. Negative affect	2.48	0.86	0.06	−0.15	0.41**	−0.06	0.22	−0.20	**(0.84)**	−0.17**
8. Job satisfaction	3.78	0.85	−0.35**	0.33*	−0.30*	0.07	−0.16	0.74**	−0.17	**(0.87)**

N_within, between_ = 495-55. Between-subjects correlations are lower the diagonal, within-subject correlations are upper the diagonal. Reliabilities are in bold and displayed in parentheses in the diagonal. *p < 0.05, **p < 0.01.

## Methods

### Design

To test our hypotheses, we conducted a quantitative daily diary study (Ohly et al., 2010). In the first stage, participants responded to a survey asking them about their demographic information and ratings of emotion regulation behavior and trait affect (i.e., control variable). After one-week, participants started to respond to a daily survey, across 9 days, in which they reported their affective states and job satisfaction. This design was appropriate to test the hypotheses because affect states are highly dynamic over time, fluctuating daily, and previous research has shown that job satisfaction varies over a daily timescale as well (Ilies et al., 2006).

### Participants

Participants were 55 professional employees of diverse organizations and occupations recruited from an MBA program at a major university in Chile. Gender of participants was 80% males, and the average age was 33.64 (*SD* = 5.10). In terms of their organizational roles, participants performed in positions of professional staff (27%), supervision or team leaders (40%), and executive managers (33%), and their average organizational tenure was 4.73 (*SD* = 4.03). The industry of the participants’ organizations were services (78%), manufacturing (13%), and consultancy (9%).

### Measures

Emotion regulation behavior was measured with the scales used by Niven et al. (2011). They asked participants about the extent to which they perform a series of behaviors to improve or worsen their feelings in general in their lives. Item examples: “I think of positive aspects of situations confronted,” “I laugh to feel better,” (4 items for affect-improving, α = 0.72), and “I look for problems in my current situation,” and, “I think about negative experiences” (3 items for affect-worsening, α = 0.83) (1: not at all – 5: many times). Affect was measured with the scales of Warr et al. (2014), which ask participants about whether they have experienced a series of affective states during the day, with the steam “Today, to what extent have you felt…” enthusiastic, joyful, inspired, active (positive affect, α = 0.84), and nervous, anxious, tense, worry (negative affect, α = 0.84) (1: not at all – 5: many times). Job satisfaction was measured with 3 items of the scale of Cammann et al. (1983), in which participants were asked to rate their degree of agreement with a series of statements such as “I am satisfied with my job” (1: totally disagree – 5: totally agree, α = 0.87). Furthermore, trait affect was also measured with 10 items of PANAS scales (Watson and Clark, 1994), asking participants to what extent they experience a series of emotions in general in their life, item examples are enthusiastic, inspired, and active (positive trait affect, α = 0.80), and nervous, irritable, and upset (negative trait affect, α = 0.83) (1: not at all – 5: many times). These variables were included as co-variables in the model estimated since previous research has shown that they are personality traits that influence job satisfaction (Judge et al., 2008). Also, the inclusion of trait affect helps control for possible confounding effects in the relationship between affect and job satisfaction. Finally, whether participants were professional employees was also included as control variable, to account for possible effects on job satisfaction associated with performing more complex tasks and have more autonomy and status in organizations (Bowling and Hammond, 2008).

### Analytical Strategy

Multilevel analysis was utilized to analyze the data with M*Plu*s. This strategy was appropriate because repeated daily measures of affect and job satisfaction were time-level variables nested in each participant (within-subjects, level-1), while emotion regulation behaviors and trait affect were person-level variables (between-subjects, level-2). First, multilevel confirmatory factor analysis was conducted to determine the robustness of the measurement model underlying the hypothesis testing (Byrne, 2012). Specifically, two models were estimated. In the first model, affect and job satisfaction measures were tested, while, in the second independent model, measures of emotion regulation were examined. This piecemeal strategy was adopted because the number of observations at the person-level of analysis was insufficient to test all the variables in a single model. Second, hypotheses were tested with multilevel structural equation modeling with observed variables, using the framework developed by Preacher et al. (2010). In the model estimated, hypotheses testing was based on 2-1-1 mediation processes, in which emotion regulation behaviors and trait affect were level-2 predictors, while affect and job satisfaction were level-1 variables.

## Results

Analysis of multilevel variance composition showed substantive within-subjects variance for positive affect, 61%, negative affect, 46%, and job satisfaction, 23%, supporting that these variables fluctuate over time. Additional analyses showed that values of skewness and kurtosis for all the measures derived the model tested minimally deviate from zero [interval of values (0.03–2.52)], which supported the subsequent confirmatory factor and structural equation modeling analyses using maximum likelihood estimation (Byrne, 2012)^1^. Confirmatory factor analysis showed excellent goodness-of-fit for the model of affect and job satisfaction, χ^2^(*df*) = 63.73(*41*), *p* < 0.05; RMSEA = 0.04, CFI = 0.98, and for the model of emotion regulation behavior, χ^2^(*df*) = 12.23(*13*), *p* > 0.05; RMSEA = 0.00, CFI = 1.00. Thus, the measurement models underlying the hypotheses stated were supported.

Results of multilevel structural equation modeling (Table 2) showed that affect-improving emotion regulation was positively related to positive affect, *b* = 0.31, *SE* = 0.12, *p* < 0.05, which in turn was positively related to job satisfaction, *b* = 0.32, *SE* = 0.07, *p* < 0.01, and a positive indirect effect of affect-improving emotion regulation, by means of positive affect, on job satisfaction was also observed, *b* = 0.10, *SE* = 0.04, *p* < 0.05. Thus, hypothesis 1 was supported.

**TABLE 2:** Multilevel SEM for job satisfaction, affect, and emotion regulation.

Variable	Positive affect	Negative affect	Job satisfaction
*Intercept*	2.68 (0.52)**	2.56 (0.69)**	1.53 (0.64)*
**Between-subjects effects**			
Professional role (0 = No, 1 = Yes)			
Positive trait affect			0.15 (0.08)*
Negative trait affect			−0.09 (0.09)
Affect-improving emotion regulation	0.31 (0.12)*	−0.15 (0.17)	−0.05 (0.08)
Affect-worsening emotion regulation	−0.29 (0.11)**	0.40 (0.15)**	0.06 (0.17)
**Within-subjects effects**			
Time index			−0.01 (0.01)
Lagged job satisfaction (t-1)			0.32 (0.15)*
Positive affect			0.32 (0.07)**
Negative affect			−0.07 (0.04)*
*Indirect effects*			
Affect-improving → Positive affect → Job satisfaction	0.10 (0.04)*		
Affect-worsening → Negative affect → Job satisfaction	−0.03 (0.02)		
Affect-improving → Negative affect → Job satisfaction	0.01 (0.01)		
Affect-worsening → Positive affect → Job satisfaction	−0.09 (0.04)*		
ICC	0.39	0.54	0.77
Deviance	1,628.09		

N_within, between_ = 287/55. Unstandardized estimates, standard errors in parenthesis. *p < 0.05, **p < 0.01.

**FIGURE 1:**
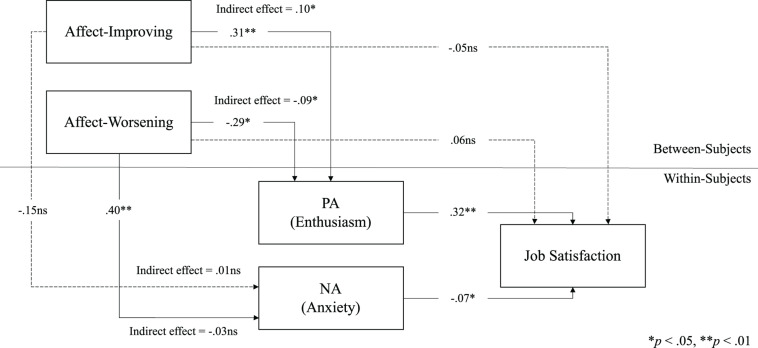
Multilevel SEM for job satisfaction, affect, and emotion regulation. ^∗^*p* < 0.05, ^∗∗^*p* < 0.01.

Furthermore, affect-worsening emotion regulation was positively related to negative affect, *b* = 0.40, *SE* = 0.15, *p* < 0.01, which in turn was negatively related to job satisfaction, *b* = −0.07, *SE* = 0.04, *p* < 0.05, but an indirect effect among these variables was not supported, *b* = −0.03, *SE* = 0.02, *p* > 0.05. Consequently, hypothesis 2 was partially supported. Although not hypothesized, results also showed a mediation process between affect-worsening emotion regulation, positive affect and job satisfaction. Specifically, affect-worsening was negatively related to positive affect, *b* = −0.29, *SE* = 0.11, *p* < 0.01, which in turn, as stated above, was positively related to job satisfaction, *b* = 0.32, *SE* = 0.07, *p* < 0.01, and a negative indirect effect among these variables was also observed, *b* = −0.09, *SE* = 0.04, *p* < 0.05 (Figure 1).

## Discussion

This study’s results supported that self-regulation of emotions is related to job satisfaction through the experience of affect at work. As we hypothesized, affect-improving emotion regulation was positively related to positive feelings while working, increasing thereby the likelihood of positive judgments toward the job and the organization. Although not expected, affect-worsening emotion regulation was negatively related to job satisfaction by reducing positive affect. Thus, affect-worsening regulation was negatively related to positive feelings, which in turn increased being satisfied at work. Furthermore, in contrast with our hypotheses, affect-worsening emotion regulation was not indirectly related to job satisfaction by negative affect. In this case, down-regulation of own feelings increased the chance of experiencing negative affect at work, which reduced the positive attitude toward the job. However, there was no direct or indirect effect of affect-worsening emotion regulation on job satisfaction.

These results highlight the benefits of affect-improving emotion regulation for the building of job attitudes in the workplace. The set of self-regulatory behaviors expressed in cognitive reappraisal and attentional deployment increase positive feelings, leading to a more positive judgment of the work environment. In contrast, the results regarding affect-worsening emotion regulation involve greater complexity. Even though the relation between affective dysregulation and negative affect is strong, the effect of the latter on job satisfaction is not, such that this path is not strong enough to describe an influence of affect-worsening on attitudes at work. Nevertheless, the influence of affect-worsening on job satisfaction is possible by the route of positive affect, because this emotion regulation strategy reduces the likelihood of experiencing positive feelings, which are substantive drivers of job satisfaction.

Therefore, we contribute to the job satisfaction literature by showing that specific emotion regulation behaviors could participate in the construction of positive attitudes in organizations. Following the EROS model of emotion regulation (Niven et al., 2011), we expanded previous research on individual differences associated with job satisfaction, by showing that cognitive reappraisal and attentional deployment are associated with the emergence of favorable judgments in the workplace, over and above other dispositions as trait affect (Watson, 2000). These results highlight that, in addition to the employees’ personality and the conditions of their work environment, how employees manage their emotions is another relevant factor to consider when assessing job satisfaction. In other words, the extent to which employees feel satisfied at work depends, to some degree, on whether they are able to sustain their positive feelings, and reduce the negative ones, while working. Thus, these findings extend our understanding of the affective rudiments of job satisfaction.

This study also contributes to research on emotion regulation in the workplace. In the work and organizational psychology, there is an important body of studies about the influence of emotional skills on work-related outcomes (Diefendorff et al., 2008; Lawrence et al., 2011). However, this research has concentrated on the notions of emotional intelligence and emotional labor. In the first case, emotion management is part of the individual abilities useful for a better adaptation to the work situation (Mayer et al., 2008); nevertheless, research about job satisfaction in this field has used general ratings of affective management, not paying attention to the specific self-regulation behavior participating in emotion regulation (Miao et al., 2017). Regarding emotional labor, this refers to the set of actions directed at managing emotional experiences according to the tasks’ characteristics or the organizational culture norms (Grandey et al., 2013). These self-regulation strategies are expressed in the simulation of feelings (surface acting) and the actual experience of them (deep acting), which has been mostly adopted to understand work-related outcomes in service jobs (Grandey and Gabriel, 2015). Therefore, our study on emotion regulation and job satisfaction expands the above streams of research because we address how specific emotion regulation behaviors, often enacted in daily life, in a diverse array of jobs, participate in the construction of job satisfaction. Furthermore, different than models of emotional intelligence and emotional labor, which are mostly concentrated on managing positive affect, our study also addressed whether emotion dysregulation, denoted by affect-worsening regulation, plays a role in the construction of job attitudes.

In practical terms, the study results point out that practitioners in the field of people management in organizations should bear in mind that employee emotion regulation is another route to promote job satisfaction. Interventions may be directed to training programs with employees for their acquisition and development of behaviors involving cognitive reappraisal and attentional deployment at work. Although affect-worsening emotion regulation does not seem to be indirectly related to job satisfaction, it is related to negative affect. Thus, training in controlling cognitive rumination should be valuable in preventing employee well-being issues expressed in negative feelings at work as well (Sonnentag, 2015).

This study has limitations to be discussed. Although the data analyzed was based on a large number of repeated measures, they were derived from a small number of participants (*N* = 55), which limited the possibility to test, in a single model, the robustness of the measurement model based on all the variables examined. The same issue led us to test the hypothesis with observed, instead of latent, variables in the context of structural equation modeling. Furthermore, the use of a diary study was an appropriate strategy to account for the dynamics of affective processes (Ohly et al., 2010). Nevertheless, this design was based on self-reports for all the variables examined. Thus, possible common-method variance issues might be present in data modeling, such that statistical estimations might be biased (Podsakoff et al., 2012). Also, because the study was based on observational data, causality among the variables studied can only be theoretically inferred. Hence, reverse causality effects cannot be ruled out. For example, job satisfaction might lead to an increased experience of positive feelings due to cognitive appraisals of the work context (Lazarus, 2001). Also, the experience of positive and negative affect at work might influence employees’ self-perceptions about how skilled they are to manage their emotions, due to affective infusion of cognition (Forgas, 1995). Therefore, future studies with larger samples of participants, using longitudinal or experimental designs, will be valuable to determine how robust the results observed here are.

To sum up, in this study, we aimed to continue and expand research on the drivers of job satisfaction from the lens of emotion regulation, showing that positive judgments about the work and the organization are a function of how employees manage their emotions. We trust that knowledge elaborated here will be informative and useful for the understanding and practice of work and organizational psychology.

## Data Availability Statement

The data analyzed in this study is subject to the following licenses/restrictions: The data utilized in this study was part of a larger dataset utilized in a previous study already published. However, there is no overlap in the variables analyzed in both studies. Requests to access these datasets should be directed to corresponding author.

## Ethics Statement

The studies involving human participants were reviewed and approved by the University of Sheffield Ethics Committee. The patients/participants provided their written informed consent to participate in this study.

## Author Contributions

All authors listed have made a substantial, direct and intellectual contribution to the work, and approved it for publication.

## Conflict of Interest

The authors declare that the research was conducted in the absence of any commercial or financial relationships that could be construed as a potential conflict of interest.

## Footnotes

1 According to Byrne (2012), absolute values above 3.00 indicate violation of normality assumption.
